# “My quality of life was not the best” experiences of Australians during the COVID pandemic: Heidi Green

**DOI:** 10.1093/eurpub/ckac131.041

**Published:** 2022-10-25

**Authors:** H Green, C MacPhail, R Fernandez

**Affiliations:** 1 Centre for Research in Nursing and Health, South Eastern Sydney Local Health District, Kogarah, Australia; 2 School of Nursing, University of Wollongong, Wollongong, Australia; 3 School of Health and Society, University of Wollongong, Wollongong, Australia

## Abstract

**Background:**

The social and economic impacts that have occurred during the pandemic can disproportionally affect those already experiencing poverty. The social determinants of health aggravate inequalities and can adversely affect wellbeing. This study aims to gain rich insight into Australian adults’ experiences of the social determinants of health and the impact on their wellbeing during the COVID-19 pandemic.

**Methods:**

A descriptive qualitative study using purposive sampling to recruit participants for semi-structured interviews, conducted via videoconferencing between March-August 2021. Thematic analysis was performed with the support of NVivo 12.

**Results:**

Participants included 20 Australian adults from various socioeconomic areas ranging in age from 21 to 65 years. Three main themes emerged from the analysis of the data: Food-related concerns; Housing outcomes; and Psychological and emotional impact. Accessing food, during the COVID-19 pandemic, for most participants who resided in low socioeconomic areas, was described as stressful and challenging. Along with the burden of food security, many participants from low socioeconomic areas expressed emotional distress in relation to securing and maintaining adequate housing.

**Conclusions:**

The pandemic has amplified existing social determinants of health experienced by those within low socioeconomic areas, particularly those who are female and from migrant communities. The wellbeing of participants from low socioeconomic areas decreased in response to their experiences and challenges with food insecurity and housing instability, highlighting the need for housing affordability strategies and funding of emergency food relief initiatives. Food access for those in areas with high socioeconomic disadvantage, can be improved to address some of the barriers associated with food security by providing supermarket meal vouchers, access to community gardens, and school food programs.

**Key messages:**

• The housing and food insecurity experienced by participants in this study during the pandemic has influenced their overall wellbeing.

• The pandemic has amplified existing social determinants of health experienced by those in low socioeconomic areas.

